# Policy‐Oriented Research on Improved Physician Incentives for Higher Value Health Care

**DOI:** 10.1111/1475-6773.12423

**Published:** 2015-11-17

**Authors:** Harold S. Luft

**Affiliations:** ^1^ Palo Alto Medical Foundation Research Institute 795 El Camino Real, Ames Building Palo Alto CA 94301

**Keywords:** Economic Incentives, physician payment, healthcare value, research opportunities

## Abstract

Policy makers (both public and private) are seeking ways to improve the value delivered within our health care system, that is, using fewer resources to provide the same benefit to patients, or using equivalent resources to provide more benefit. One strategy is to alter the predominant fee‐for‐service (FFS) economic incentives in the current system. To inform such policy changes, this paper identifies areas in which little is known about the effects of specific incentives (FFS, salary, etc.) on the two components of value: resource use and quality. Specific suggestions are offered regarding research that would be informative for policy makers, focusing on fundamental “building block” studies rather than overall evaluations of complex interventions, such as accountable care organizations. This research would better identify critical aspects of the FFS model and salary‐based payments that are particularly problematic, as well as situations in which FFS or salary may be less problematic. The research would also explore when alternatives, such as episode‐based payment might be feasible, or simply be hypothetical solutions. The availability of electronic health record‐based data in various delivery systems would allow many of these studies to be accomplished in 3–5 years with budgets manageable by public and private funding sources.

This paper suggests a series of research questions to help improve incentives for higher value health care delivery. In this paper, “higher value” means health care that either uses fewer resources to provide the same benefit to patients (as they perceive it) or uses equivalent resources to deliver more benefit. Other papers in this collection address theoretical concerns and opportunities for improving economic incentives, as well as the current evidence on existing innovative approaches.

The term “policy‐oriented research” is used advisedly, and broadly. As seen in controversies about the Affordable Care Act (ACA), when the evidence is limited or nonexistent, policy tends to be driven by beliefs rather than science. Debates about how to achieve higher value health care are likely to continue. The goal of this paper was to highlight some issues around improving economic incentives where much more research is needed to fill in knowledge gaps. It is relatively easy to propose solutions to enhance value, but much more difficult to know whether they will work in the real world. A well‐designed study demonstrating that a specific approach works well for a few procedures will not answer the question of whether it is broadly applicable. In contrast, research demonstrating how crucial problems are associated with a proposed solution or that the intervention failed to achieve its goals in an “ideal” setting should give policy makers pause. Some of the research suggested is in the latter realm—where a well‐executed study can demonstrate that a “promising” use of incentives may be problematic or not ready for “prime time.” Other research is intended to sharpen our thinking about how we think about economic incentives and their application.

Policy changes can occur at the national level through legislation, regulation, or enforcement, or at the local level, as organizations anticipate or react to changes in their marketplace and business environment, which are impacted by national policy. This paper focuses on the impact of economic incentives on physicians as they take care of patients.^1^ Physicians are sometimes paid directly by insurers (and patients). Increasingly, however, physicians are compensated by yet another intermediary, which may be a medical group, hospital, or through a less formal (nonemployment) arrangement with an Accountable Care Organization (ACO) or HMO.

The U.S. health care delivery system is undergoing substantial changes. The ACA and the replacement of the sustainable growth rate (SGR) formula under Medicare are fundamentally changing the way Medicare pays providers. Secretary Burwell has committed to replacing the majority of fee‐for‐service (FFS) payments with alternative approaches within a short time period. Private payers are shifting risk to providers through shared savings plans. These changes implicitly or explicitly include financial risk for services delivered by others, and potentially accountability for quality metrics that may reflect the efforts of multiple clinicians, organizations, and the patients themselves. New entities, such as accountable care organizations, are being created to serve as intermediaries between the payer and the clinicians. These organizations will need to develop their own approaches (policies) on how to compensate their clinicians. Existing medical groups that had FFS revenue streams are reassessing how they compensate their clinicians. It will take years, however, to assess the impact of such changes; implementation is slow, existing contracts may run for several years, and even first‐round impacts on providers, medical technology firms, and potential market entrants are uncertain. Second‐ and third‐round effects as those players respond to changes are unpredictable.

Data from prior to the ACA reflect the incentives and structures of that era; data currently being produced reflect a system in transition. To be of value for informing policy, research on incentives should be interpreted with care. Evaluations of major changes such as ACOs are not proposed here, both because they are already “on the agenda” and because such overall evaluations are not designed to assess component features of various incentives. At best, they will be “high‐level” assessments of whether Medicare's ACOs achieve the targeted savings and quality metrics, or perhaps whether certain types of organizations seem to do better than others. The problem is that most such data will be “noisy,” in that different organizations applying the same incentive changes will achieve differing outcomes due to unmeasured factors related to their history, leadership, local market environment, contract timing, etc.

Ideally, we would be collecting data on how organizations (both old and new) are using, modifying, and developing incentives for clinicians as their external environment is changing and how well those new incentives are achieving their goals. Such a research project, however, is beyond the scope of the studies envisioned in this paper. It would require a very extensive effort reaching out to organizations, asking them to share what they are doing, and planning on doing, in a highly uncertain and fraught environment. This information is often very sensitive, both because it could reveal important business strategies, but also because it may expose internal stresses as some clinicians within the organization gain and others lose. For such research, informed consent (and potential organizational withdrawal *during* the project) makes the usual IRB approvals look simple. The Center for Studying Health System Change previously funded by the Robert Wood Johnson Foundation was probably the closest model for this type of work, but even they had limited access to the inner workings of organizations (Mathematica Policy Research 2013).

The gold standard in science, the randomized controlled trial, is rarely an option for studying comprehensive effects of incentives in health care delivery. For example, even the classic RAND Health Insurance Experiment of the 1970s randomized patients to different insurance coverage, not providers to deal with different payment structures (Newhouse et al. 1993). It could answer questions about patient‐focused incentives on patient demand for care, but not how providers would react if all or most of their patients face new copayments, let alone how manufacturers would alter their business models if patient copayments changed.

The large datasets available for many modern studies can yield statistically significant findings of little clinical (or policy) import, or they may fail to model appropriately critical factors. The research suggested here often harks back to classic hypothesis testing, but in a policy‐relevant manner. It is not very useful to demonstrate with a high degree of statistical certainty a policy irrelevant finding, for example, that patients of physicians paid by salary use fewer resources than those whose physicians are paid FFS. The interesting question is whether this is always the case (assuming the tests are powered appropriately) and, if not, what characterizes situations in which there is no meaningful difference in resource use. The focus of this paper was therefore on areas in which little is known, where one or two research findings may have an important impact on our thinking, and how such studies should be designed to avoid misleading policy implications. Many of the studies suggested are “illustrative” in that they draw upon a particular clinical condition or care process. Their failure to show an effect (if appropriately designed and powered) should set aside or call into question a hypothesis. Alternatively, careful examination of the reasons for their findings should yield important insights.

This paper begins with a quick overview of its basic assumptions and focus to set the framework for what is being proposed. It then discusses some critical research design issues underlying the suggestions for potential research projects. The body of the paper has three sections exploring various aspects of economic incentives for physicians and how they may be explored empirically to inform policy. The first of these, “Beyond Caricatures of Incentives,” encourages researchers (and hopefully policy and organizational change advocates) to think about incentives in a more precise and nuanced fashion. This includes (1) distinguishing FFS per se from FFS payments that embody financial conflicts of interest; (2) clarifying what FFS and “straight salary” actually mean; and (3) focusing on everything physicians order—not just the services they provide directly. The next section, “Beyond Just Paying for ‘The Visit,’” introduces a third compensation model, episode‐based payment, and research needed to better understand when such an approach may increase value. The third section, entitled “Transitioning from the Current Set of Incentives,” focuses on practical issues related to policy implementation at a national and organizational level. Each section begins with a brief summary of the incentive or policy area; identifies various aspects in which more research needs to be done; and suggests, in italics, studies to shed light on the issues. A concluding section offers a brief roadmap for moving forward.

AHRQ commissioned this paper to encourage proposals for potential research that it and other funders might support. It is not intended to be a thorough review of the field—that is addressed in the companion papers. Nor is this a “research agenda” that has been discussed and vetted with a broad audience. Rather it reflects the author's suggestions for work that could inform our collective understanding of incentives for physicians. Each research idea roughly meets two criteria: (1) it could be undertaken in 3–5 years with data that exist somewhere, albeit rarely in public‐use data files, and (2) it could be accomplished with budgets within the scope of federal research agencies and private foundations. Those two criteria exclude an effort to really understand how organizations are internally changing to adapt to the new policy environment.

## Underlying Assumptions and Focus

As pointed out in the other papers in this series, there are several key problems with FFS incentives for physicians (see Conrad 2015; Berenson and Rice 2015). Patients do not fit the theoretical model of independent economic actors deciding whether the cost of a service is worth the expected incremental health benefits. Patients rarely have the technical expertise to reach a diagnosis or execute a treatment and thus rely on physicians as agents. Various types of health care, moreover, raise different challenges: (1) preventive care is often misvalued because people do not deal well with probabilities and future outcomes; (2) acute life‐threatening care is very expensive, often requiring quick decisions when a patient may not be in a position to rationally choose among the options; (3) chronic care often requires a set of clinicians with specialized skills who may not communicate with each other or share incentives. A fourth category, minor self‐limiting acute problems, presents fewer issues and is less important from a policy perspective (Luft 2008). Preferences for health and various interventions vary across individuals and over time as life circumstances change. Moreover, care is delivered by people, not machines or pills; a specific problem may on average be better managed surgically than medically, but information on the performance of the available surgeons and medical specialists is generally lacking (Landon et al. 2003; Hussey, Luft, and McNamara 2014). Given these realities, an optimal one‐size‐fits‐all solution to value‐enhancing incentives is unlikely.

Identifying the optimal set of services to be provided in a given situation may be too lofty a goal. Instead, our goal should be designing incentives and structures more (rather than less) likely to increase value in specific classes of situations. That is, we should seek not a recipe book with the “best” treatment for each condition, but ways superior to the current FFS model in bringing increased value to each patient. Indeed, “what the patient needs” may often need to be provided by various physicians and other clinicians, as well as support services. It may be better to place the responsibility for coordinating care on an organization, either for all the care needed for a year or (much) longer via capitation, or for specific problems, such as treating and managing cancer with an episode‐based payment. Shifting from FFS to such models makes sense conceptually, but it raises complex issues regarding clinical risk adjustment, financial risk bearing, accounting for patient preferences, and implementation in the absence of comprehensive data. These are issues for another paper. As organizations take on such responsibilities, however, they need to consider how to compensate their physicians—the focus of this paper. Importantly, incentives directionally superior for some situations may be inferior for others. Much of the research suggested herein focuses on examining when certain incentives are better than others.

Value is often conceptualized as health benefit relative to resource use, but this notion does not address whether these (conceptual) metrics should reflect a societal perspective or individual patient preferences. The latter may place a high value on services that offer a feeling of being “cared for” yet yield no detectable improvement in health that would be included in a societal perspective. Nor is it clear whether charges, actual payments, cross‐subsides, etc., should be accounted for in the resource use calculations of various policy makers. Clearly, assessments informing national policy might use different approaches than those informing payment changes within an organization. Even setting aside those issues, research attempting to assess differences in value associated with different incentive approaches is a task well beyond the scope of this paper.

Insurers, public programs, or patients may pay individual providers such as physicians directly, or they may pay organizations that in turn compensate individuals as employees or contractors. One needs, however, to get beyond those general models. Most payers rely on FFS, but Medicare also uses forms of bundled payment for ESRD providers and DRGs for hospitals. As an example of intermediary organizations, public hospitals often receive FFS Medicare and Medicaid payments, but their staff physicians are salaried. Payers also may rely on organized systems to take care of populations, for example, HMOs, with risk‐adjusted premiums or the Veterans Health Administration with an annual budget. Such systems can use various approaches to compensate the individual providers associated with (or working for) the organization.

In general, with FFS one can assess whether a specific service was provided, and perhaps whether this was consistent with specific clinical guidelines. Such guidelines, however, often leave substantial discretion to the clinician because the true value of a service to a specific patient may depend on a wide variety of unmeasured, or unrecorded, factors. Given suitable risk adjustment, one could assess the value provided a sufficiently large population, but it is often difficult or impossible to determine whether a specific service added value to a given patient. Thus, we must typically rely on indirect measures of whether changing incentives improves value.

Not all incentives are financial—most clinicians want to provide the best care they can for their patients. When paying their clinicians, organizations may combine incentive and monitoring systems. While internal incentives (especially nonfinancial ones) may be critical and are touched upon in the paper by Berenson and Rice (2015), there is not much that policy makers can do to affect how such incentives are deployed. As Roland and Dudley (2015) point out, public reporting and other monitoring of quality and value at a system level may have important effects on what and how incentives are used internally and on overall performance. Such direct measurement and reporting, moreover, may *only* be possible at the organizational level because the small number of cases seen by most individual physicians makes the data statistically unreliable.

Accountable care organizations are an important addition to the traditional FFS versus HMO dichotomy. ACOs typically include physicians located in solo and small group practices primarily paid on a FFS basis. Payment to the ACO is typically a blend of FFS with some sharing of savings, but the larger patient base for which the ACO is accountable allows ACOs to have reasonably valid organizational quality metrics. Early results from ACOs are beginning to be published and are somewhat encouraging (Casalino 2015; Nyweide et al. 2015). It is important to remember, however, that ACOs are voluntary associations, they are learning how to deliver care in new ways, and they are already under significant scrutiny.

Our research agenda attempts to address the “apple picker” analogy proposed by Kronick, Casalino, and Bindman (2015) in the context of payment to *physicians*, rather than to *systems*. A substantial literature compares the performance of HMOs and FFS settings (Luft 1980; Miller and Luft 1994, 2002). That work indicates that the incentives used in HMOs, in combination with the physicians who chose to be in those systems, results in high‐value care for their voluntary enrollees. What is less clear, however, is whether the same results would arise if one were to change by fiat the payment models for the majority of US physicians and patients not in such systems. Moreover, absent legislation, HMOs will continue to be voluntary. Thus, we focus on incentives that can impact those physicians who are still paid largely on a FFS basis, and on compensation models that may be implemented within systems.

Whether or not apple pickers care about anything other than their pay, most physicians do. Although FFS may reward delivery of more services that are compensated, that does not mean FFS drives physicians to provide unnecessary services. Rather, in the absence of value‐based reporting or alternative organizational structures, FFS may support a wide range of practices (Luft 2012). Indeed, without newly provided data, some physicians may not even know their practice style is outside the range of what is supported by the evidence. With some tweaks, however, FFS might be an effective modality for some situations in a toolkit of payment approaches. The research suggested is intended to explore this possibility.

## Research Designs

The focus of this paper was on new ideas for research that would return policy‐relevant findings within 3–5 years and are likely to fit within current research funding realities in both the public and private sectors. This favors observational studies, often using settings with excellent data and differing incentive structures or, better yet, differing incentive situations within the same organization. This often requires exploring the details of incentives within organizations. For example, the “classic” HMO is exemplified by the Kaiser Permanente plans in California that receive a premium and transfer a capitated amount to their medical groups to care for a defined population. Within the medical group, physicians are essentially paid a salary with incentives for meeting particular quality and service goals. In Group Health, another HMO, a similar arrangement exists for some physicians, but outside the Seattle area the HMO contracts with physicians on a modified FFS basis. Group Health also runs a preferred provider organization that looks like an insurance plan. Health Partners in Minneapolis‐St. Paul pays its core medical group a capitation amount, but the group's physicians are essentially compensated based on productivity. The Palo Alto Medical Foundation (PAMF) in California receives most of its revenue on a FFS basis but has significant capitated HMO contracts and shared savings (ACO‐like) arrangements with insurers and employers. PAMF's physician group is paid proportional to physician work (i.e., excluding ancillary service revenues). Careful examination of these organizations reveals important incentive differences that could be used in certain research designs. Each of these organizations, moreover, participates along with many others in the Health Care Systems Research Network (formerly the HMORN) with the ability to share comparable EHR‐based data for research.

Ideally, researchers would have measures reflecting the long‐term effects of various clinical interventions on patients, focusing on dimensions that generally matter to people and how different individuals value those dimensions. Such measures would include relevant perspectives on economic sustainability. Not being in such an ideal research world, however, means settling for proximal measures while looking for unintended consequences. At times, this may require focusing on relatively short‐term cost and quality indicators, for example, within an episode of care.

The episode of care offers a potentially valuable “unit of observation” for researchers to pull together all the services involved associated with a specific patient problem.^2^ Ideally, quality metrics would be applied to the episode. Because episodes deal with how a specific condition is addressed once it occurs, risk adjustment for differential occurrence across populations is less critical. Knowing the patient's other clinical conditions allows statistical controls for comorbidities that either complicate the care of the “target” problem or, conversely, allow it to be managed with other problems during the same visit. Episodes, however, are not suited to assessing preventive efforts intended to keep a problem from arising.

Some research may be interventional, that is, purposefully changing the incentives for a set of providers and seeing what happens. Other changes may support quasi‐experimental designs. Much may be learned, however, from well‐designed studies using comparative observations from settings with different incentive systems—as long as one is wary of potential confounding reasons why those settings have different incentives. Thus, referring to the examples above, comparing overall resource use and quality by physicians at Kaiser and PAMF would be of limited value because *both* the physician payment incentives *and* the organizations differ. The organizations may have attracted differently oriented physicians and possibly patients with differing expectations for the use of services.

Physicians are not randomly assigned to compensation models, so one cannot refute the possibility that physicians with specific clinical decision‐making orientations differentially gravitate to FFS and salaried settings. To inform policies regarding incentives, studies should be designed to be convincing to skeptical physicians and policy makers, perhaps by specifying in advance the pathways likely during different types of episodes and how different payment models could affect each pathway. For example, Chung et al. (2015) showed that Medicare's expansion in coverage for annual wellness visits (AWVs) not surprisingly led patients with FFS coverage to have more wellness visits. Using detailed information in the electronic health record, they could assess the differential impact this had on physician activities that were time intensive versus not or were included in quality metrics versus not. They also compared the effects for these Medicare FFS patients with comparably aged patients having Medicare HMO coverage or employer‐based FFS or HMO coverage unaffected by the AWV policy change. One would not expect everything to change in the same way, and the pattern of results indeed aligned with predictions based on incentives.

Observational research is best done with nuanced studies having different hypothesized incentive effects for different conditions, services, or situations—ideally not everything should move in the same direction. Incentives may also affect what data are captured and how they are coded. If payment depends on certain documentation, new payment incentives may simply alter documentation, not practice, so the researcher should attempt to validate findings with multiple data sources subject to different biases. The next section delves more deeply into details about incentives under FFS and alternative approaches that can inform such research.

## Beyond Caricatures of Incentives

The theoretical work on incentives associated with FFS appropriately deals with issues abstractly (Conrad 2015). The caricature is that, everything else being equal, FFS rewards physicians for providing more services rather than for delivering value, resulting in too much care. At the other extreme, straight salary is seen as offering no rewards for increased effort or higher quality. In practice, everything else is rarely “equal,” and while some evidence can be found to support each caricature, that evidence may not be generalizable. In this section, we highlight three particular areas for further examination: (1) FFS per se versus FFS Embodying Financial Conflicts of Interest; (2) What do FFS and Straight Salary Mean?; and (3) Focusing on What the Doctor Ordered.

### FFS *per se* versus FFS Embodying Financial Conflicts of Interest

It may be fruitful to explore whether the “problem” is FFS per se, or specific aspects of FFS, such as when the amount paid is so great as to make the physician a conflicted agent for the patient. This can occur when a physician has an ownership stake in a service he or she is providing or ordering, such as an imaging center. It can also occur when the existing physician payment model includes revenues for things aside from the services actually rendered by the physician. For example, oncologists are sometimes paid a relatively low fee for the provision of their services but are allowed to retain the markup on the chemotherapy agents they provide (Newcomer 2012). Both CMS and the American Society for Clinical Oncology have proposed alternatives to this standard model. If the “fee for chemo” model incentivizes additional rounds of therapy or more, rather than less, expensive agents, then the problem may lie in that particular aspect of the FFS pricing. If it appears that FFS is particularly problematic when accompanied by financial conflicts of interest but is more benign when those conflicts are eliminated, then the focus might be on eliminating the conflicts, not replacing FFS.

- Research might examine the number of rounds of chemo and the specific agents used (controlling for cancer type and stage) by different oncologists whose income is based on (a) straight salary; (b) work RVUs; or (c) total billings.^3^ If FFS per se is the problem, the number of rounds and chemo costs for patients of physicians in categories b and c should be similar, and be higher than those in category a. If the financial conflict of interest is the problem, the rounds and chemo costs should be much higher for patients of physicians in category c, but might even be similar for those of physicians in categories a and b.
- Such analyses must control for type of cancer, patient age, comorbidity, etc. and focus on situations in which there is little evidence of improved survival or quality of life with additional rounds or with agents having a greater profit margin.

There are other arenas in which the existing FFS model involves more than just physician fees. Unlike the case of oncologists, who both order and provide chemo, imaging is interpreted by radiologists but is usually ordered by other physicians. Due to high capital costs, advanced imaging tests (CT, MRI) typically have facility fees well above their marginal cost, so higher use can yield substantial profit to the owner of the equipment. Interpreting images is complex, so radiologist reports may reflect that uncertainty and legitimately encourage additional scans. Radiologist work effort is relatively constant per image, so compensation based on wRVUs may not have incentives all that different from those of salary. (Unlike Federal judges appointed for life, if there isn't enough work to be done, even salaried physicians may become unemployed.) In theory, the requester of the imaging study, whether paid FFS or salary, merely wants information valuable in treating the patient.

If the ordering physician has an ownership stake in the imaging facility, however, a financial conflict of interest arises from the profit associated with increased facility use (Hillman et al. 1990; Levin and Rao 2011; Schneider et al. 2012). If the radiologist is also an owner of the facility, s/he has additional incentives to encourage repeat scans, but these likely reflect the ownership stake, not the FFS payment.^4^

- Research might explore, on a condition‐specific basis, whether the likelihood of initial imaging orders by physicians differs based on whether the ordering physicians and/or the radiologists have ownership stakes in imaging facilities.
- Likewise, research should explore whether the number of scans done per order and per episode, say of acute back pain, differ. Theory would suggest radiologist ownership of the facility should affect the number of scans per order, but not the likelihood of a request. Ownership of facilities by ordering physicians should have a greater impact on initial requests, and it might also affect scans *per order* even if the radiologists have no ownership stake.
- From a policy perspective, one should know whether ownership is more important than how the ordering physician is paid for his/her services. Such research requires good data on ownership and the implicit financial incentives for “nonowners.” One potential source of such information might be teaching hospitals with different faculty and departmental compensation arrangements.

The standard critique of FFS is that it rewards volume provided, not value to the patient. Outside of medicine, however, most goods and services are sold using a FFS‐like model. What is different in health care is that (a) the patient usually pays only a fraction of the cost and (b) the patient often needs physician advice about whether a service is “needed” (Conrad 2015). The extensive literature by Wennberg and others on service use variations suggests that when criteria are very clear (hip fracture) there is little geographic variation among providers paid FFS, but when clinical evidence of value is lacking or conflicting (hip replacement), substantial variability is observed (Wennberg and Gittelsohn 1973; Welch et al. 1993). Some see FFS payment as inducing demand, but that is too simplistic—one would expect uniformly high rates of “discretionary” cases across all surgeons paid FFS (Mehrotra, Dudley, and Luft 2003). In fact, FFS payment seems able to sustain almost any level of use (hence the variability observed within Medicare FFS). Contrast this with the strong incentives from having an ownership interest in a facility. Variability per se does not mean patients in high‐use areas are not receiving significant benefits; for this we need good measures of quality, including patient‐reported outcomes. The RAND work in the 1990s on various surgical and diagnostic procedures found little relationship between rates of utilization and appropriateness of care (McGlynn 2013).

- Research should be undertaken to better understand what accounts for the variability in recommendations in addressing patient problems by physicians in the same specialty compensated in similar ways, for example, FFS or salary. Such work must take account of how patients work their way through the health care system, from initial diagnosis to definitive treatment.
- Does it matter if the situation is one in which the initial diagnosis is made by a generalist who can then refer to any of several types of specialists? For example, a patient with back pain might be referred to a physical therapist, physiatrist, or orthopedic surgeon, versus one in which the diagnosis needs to be made by a specialist who may or may not have a preferred treatment approach?
- How much of the variability reflects patient preferences (an aspect of value) rather than physician recommendations?

Policy makers often focus on economic incentives for providers to deliver too much care; for example, because “surgery is what surgeons do,” they always encourage more surgery. Less attention has been paid to economic incentives discouraging the use of appropriate services (McGlynn et al. 2003). Some preventive activities merely involve discussions with patents, rather than ordering tests or giving immunizations. If FFS does not adequately compensate discussions, such as about preferences for end‐of‐life care, they may be underprovided. A recent study suggests Medicare's decision to pay for AWVs substantially increased the delivery of such services (Chung et al. 2015). The proposed new Medicare payment for end‐of‐life care discussions offers an opportunity to extend this work.

- When FFS does not provide compensation, there are typically no claims generated, but this changes when coverage is provided. This may lead to greater changes in documentation than actual practice. Researchers need EHR and patient‐based survey data and designs robust to independent changes in guidelines and coding to distinguish changes in actual use versus how it is recorded. The results of such studies may help policy makers increase their confidence in the effects of coverage changes.

### What Do FFS and Straight Salary Really Mean?

Theoretical discussions (apple pickers vs. federal judges) often contrast FFS (simple piece‐rate) and salary (pure time) compensation schemes. In practice, most compensation models have countervailing performance criteria. For example, if physicians (or anyone else) are paid hourly salaries, there is an expectation they may work a little less hard per hour than if paid per service or piece produced. Organizations may counter this incentive by setting expectations for images reviewed per hour or appointments scheduled per week. Such performance criteria, however, may be more effective at getting everyone within the organization to the same standard than in improving overall value, that is, productivity controlling for quality. There is relatively little research on the performance implications for physician work of salary‐based payment, or for different ways of accounting for “pieces.”

Paying anesthesiologists by time is quite common, and probably not problematic incentive‐wise because they have relatively little control over the length of the operation. Likewise, paying a radiologist FFS to interpret a scan may not be problematic—assuming someone else orders the scan and the radiologist has no ownership interest. Whether paying the surgeon for a procedure is “problematic” may depend on whether he or she influences whether the procedure is done. The question of whether the payment model leads physicians to encourage a patient to have something done is set aside for now to focus on the details of how performance is monitored and payments structured.

- Research should explore what countervailing performance metrics are used when physicians are salaried and whether the organizations feel such metrics are working well. For example, when face‐to‐face visits dominated primary care, it could be sufficient for an organization paying its physicians a salary to simply monitor or control appointments. How communication substituting for visits (phone, e‐mail) is now accounted for in such frameworks is worth exploring.
- Research should then be undertaken on whether and how productivity differences associated with payment models (and their various countervailing metrics) impact quality. For example, when comparing surgeons in FFS and salaried settings, how many procedures (adjusted appropriately for complexity) do they do per week? How many hours do they spend in practice? Is their time per procedure different? Is the frequency of surgical and anesthesia complications associated with the average “speed” of the surgeon?
- Do other methods for improving productivity, such as standardized design processes, increase throughput? What effect have these methods had on quality?
- Are these quality and productivity improvement methods easier to implement if surgeons are paid FFS or salary?

Discussions about FFS (and implicitly its alternatives) rarely distinguish between payments made to the physician and other payments that are intertwined with the service.^5^ Most surgical procedures have a facility fee for the operating room or ambulatory surgical center. Many also have fees for the anesthesiologists' time. For various policy discussions, it would be helpful to know the components of such direct FFS payments within the Medicare and Medicaid programs. This would provide a lower bound on the dollars directly linked to FFS payments; for example, although anesthesiologists are paid on the basis of time, their services are driven by payments to surgeons.

- FFS claims can identify services nearly always linked to certain physician activities, for example, to specific surgical procedures or images ordered. For each service (or groups of services), what fractions of total payments are attributable to (1) work effort of the “submitting/lead physician”; (2) work effort of other physicians; (3) malpractice costs; (4) facility costs; and (5) other costs?
- Do the proportions attributable (within specific procedures) to these components vary across payers (Medicare, Medicaid, private insurers)?
- What proportion of all payments by these payers are contained within the selected group of procedures?
- Do empirical business models of physicians' practices, for example, accounting for equipment, repayment of medical school loan, include these factors in a way that is “good enough” to assess the implications of major payment changes?
- Answers to such questions will help policy makers assess how payments might be affected by certain policy changes, and how such changes would affect various types of physicians.

### Focusing on What the Doctor Ordered

Discussions about physician incentives typically focus on their personal direct or indirect compensation. Physician income is roughly 21 percent of health care expenditures, but physician “orders” account for another 66 percent (Sager and Socolar 2005). Absent an ownership stake, FFS is generally incentive neutral regarding services delivered *by others*, for example, lab tests and imaging, which provide information to the physician in making a diagnosis. Such tests can reduce a physician's uncertainty, but they may be clinically unimportant if monitoring symptoms over a relatively short period of time provides comparable information. The physician, not the patient, is principally aware of, and affected by, this uncertainty.

Talking with the patient about whether to order a test takes time, but fees are generally not proportional to time. Thus, wRVU‐based payment may discourage physicians taking the time to discuss the risks and benefits of a test. That time could be used to address a different problem (possibly warranting a higher RVU code) or to see a different patient. Contrariwise, a physician discussing why a test should not be ordered may feel justified in coding a visit as complex and generate more income to offset the extra time. For physicians paid a salary, similar time pressures may impinge via organizational standards on the number of patients seen in a day.

- Is the likelihood of a test order conditional on a presenting problem related to physician time pressure during the visit and does this vary by how the physician is paid? EHR systems often have “time stamps” associated with every action, that is, when physicians “open” and “close” a patient's chart in the examination room. Linking this to when the appointment is scheduled (or better, when the physician's *next* patient is scheduled to be seen) indicates whether the physician was running late. If time pressures matter, then patients seen when the physician is running late may be more likely to have a lab or imaging test ordered, controlling for patient conditions, etc.
- Patients with colds sometimes ask for unnecessary antibiotics; do uncompensated time pressures appear to prevent some physicians from resisting such requests?^6^ The answers to such questions will help assess whether payment approaches that minimize physician time have unanticipated consequences in certain circumstances.

A FFS environment overcompensating “things that can be done quickly” is problematic, as are salary systems with inappropriate performance expectations. In theory, compensation should be comparable across all the activities a type of physician does. The wRVU values for procedures, however, typically get set soon after their introduction. Although procedures often become faster to execute, the wRVU weight is rarely readjusted downward. Communications between patients and physicians now occur via secure e‐mail, sometimes without further contact. If messaging generates no wRVUs, such communication may be disincented. Some insurers are beginning to pay for certain types of messaging but others are not, creating an opportunity to assess the impact of such payment. With salaries, messaging may be discouraged if only face‐to‐face visits count in meeting organizational performance. Messaging, moreover, often occurs outside of the physician's usual office hours, but this infringement on one's “home life” may be less problematic for physicians paid wRVUs for messaging than those receiving no compensation for messaging because they are salaried. Research into these issues must rely on data from EHR systems, looking more deeply into “what happened” during, before, and after a specific visit or encounter, preferably with reasonable quality metrics.

- Is payment for messaging associated with increased messaging by physicians and decreased use of face‐to‐face visits or other expensive services?
- How do organizations compensating physicians by salary account for messaging?
- Do different compensation approaches have different implications for the care delivered?

## Beyond Just Paying for “The Visit”

Much of the previous discussion has focused on whether the mode of physician compensation for a specific visit, for example, wRVU versus straight salary, affects what *they* order and the resulting quality of care. One can use an episode‐based *analytic* focus to examine what services are used to treat an acute problem, such as back pain, a complex and typically expensive problem such as newly diagnosed breast cancer, or the full‐year management of a patient with a chronic condition, such as diabetes or hypertension. With comparable EHR data, one can examine the services used for such episodes of care, even for services without a claim, for example, secure messaging. For some episodes, quality metrics can reflect outcomes or adherence to specific clinical guidelines, for example, the prescription of appropriate medications. This section goes beyond the simple FFS/salary dichotomy to two potentially important policy options—episode‐based payment and providing information.

### Episode‐Based Payment

The bundled, or episode‐based, payment approach is under discussion for selected procedures, such as hip replacement in which the payment covers professional services, facility costs, and the implant. Some proposals for episode‐based payments are intended to adequately compensate physicians for appropriate care, but not for care not deemed to be appropriate (de Brantes, D'Andrea, and Rosenthal 2009; de Brantes, Rosenthal, and Painter 2009). Initial results of bundled payment experiments have been mixed and interpretations of those findings vary (Pham et al. 2010; Hussey, Ridgely, and Rosenthal 2011; Mechanic 2011; Williams and Yegian 2014). While most of the focus has been on bundling payments from a payer to a set of otherwise independent providers, the approach could be adapted for compensation *within* an organization, for example, an ACO or a medical group.

For surgical procedures, identifying the “lead physician” is relatively easy. In other situations (e.g., management of diabetes), episode‐based methods of payment shift to an “attributed physician” some financial risk for services ordered and the quality of care delivered (or, worse from a physician perspective, even obtained by patients without the approval of the “attributed physician”). Studies have explored the implications of various “attribution” methods based on claims data (Sandy, Rattray, and Thomas 2008; Mehrotra et al. 2010). Understanding the financial and other implications of various attribution approaches is critical in moving from a theoretically attractive option to technically and politically feasible policies. Large organizations such as HMOs can take on significant amounts of risk. The experience of new ACOs suggests that accepting risk is not always attractive, and for individual (or small groups) of physicians, accepting even limited risk via bundled payments is likely to happen only with substantial evidence of how it will play out.

- Research should explore how well various attribution methods align with what physicians see as being “fair,” to predict the acceptability of various techniques.
- In some settings, patients have an identified PCP accountable by the organization for their care in terms of various metrics. How do various “claims‐based” attribution approaches align with the “assignments” noted in the patients' electronic health records?
- How sensitive are the attributions of various approaches to collaborations among PCPs, for example, when two informally share a patient panel?
- How sensitive are the attribution methods to the number of years of data, and are there ways to account for when a patient implicitly or explicitly switches physicians?

Perhaps more important than the attribution issue is the definition of the services for which the physician is implicitly being held responsible. (This is far less an issue in terms of for what an organization, for example, an ACO, should be responsible, but it may be relevant with respect to out‐of‐area costs.) Some argue that PCPs should be accountable for all services needed (or used) by their patients. If a PCP does not offer ready access for urgent problems, it seems reasonable to attribute to that PCP the patient's visit to the emergency room (ER). From the perspective of what PCPs would perceive as being fair, however, should not one differentiate ER visits for worrisome gastrointestinal symptoms from those for motor vehicle accidents? Visits for stroke, which might be prevented through effective blood pressure control, might be in a “gray area.” This touches on two issues: (1) the degree to which the PCP should be held clinically accountable for the need for care and (2) whether the services were delivered or approved (ordered) by the PCP. A PCP should “own” the ER costs if she told the patient to go there after a phone consultation, but she might not feel it fair to be “charged” if a patient ignored available 24/7 advice lines and an urgent care center.

- The proportion of total expenditures in various categories of care, for example, physician services, labs, imaging, etc., is known in the aggregate. Research is needed, however, on the proportion of services (and costs) of various types, for example, *other* physician services, lab, imaging, and facility fees that would be attributed to specific physicians (PCP vs. specialist) for an episode “ordered” by that physician versus obtained by the patient without a specific order by the attributed physician. Such information is necessary for those physicians who would be held responsible under bundled payment to know what proportion of the episode's cost is essentially out of their control.
- This work should also attempt to categorize such costs by the degree to which physicians feel the services *should* be the responsibility of the attributed physician.

Episode‐based payment shifts financial risk to physicians. The wide variability in tests and procedure use *across physicians* is well known. Less well studied, however, is the variability in costs *across patients* seen by, or attributed to, a specific physician. Such (possibly random) variation may affect physician willingness to accept an episode‐based payment that is a fixed amount per case, albeit potentially with some risk adjustment. Even if a physician has below‐average resource use, and over several years could benefit financially under a bundled payment model, the risk of a financial shortfall in a given year may be a barrier to willingly accepting bundled payments.

- Research is needed to understand the variability in episode‐based costs across patients seen by individual physicians, or small groups of similar physicians, for example, a small cardiology group. Given a specific number of patients approximating those likely to be seen in a year and plausible sets of services for which the physicians might be held accountable, for example, perhaps excluding inpatient episodes, how often would random variability lead to “losses” greater than a certain percentage or amount?
- Research is also needed on barriers and facilitators to physician acceptance of payment models that involve risk bearing. What types of reinsurance or one‐sided gain‐sharing models could win acceptance of such approaches by providers?
- Across various ACOs and similar organizations, are some governance structures more conducive to alternative compensation methods?

Global fees that include pre‐ and postoperative visits have long been used for certain surgical procedures. If expanded to include services needed beyond those delivered by the surgeon, this becomes an episode‐based payment. These approaches could be quite problematic if the surgeon also decides whether the procedure is needed. If, however, patients first met with a neutral expert (i.e., someone paid hourly) to discuss the risks and benefits of the procedure, the surgeon and his or her team could be paid a fixed amount for the intervention and follow‐up care.

- Research is needed on the feasibility of separating decisions *to enter into care* from the decisions *on how to provide it*.
- Does using such a “unit of payment” allow one to focus more effectively on clinical and patient‐reported outcomes?
- How does this approach relate to the level of evidence available to guide patient decision making? Such research would identify various decision points along a typical patient trajectory of care. For what episodes are the data and tools available to help patients make such decisions?
- If researchers begin with conditions or interventions that appear likely candidates for such payment models and nonetheless identify substantial logical or logistical problems in such “ideal cases,” policy makers could reject this as an attractive near‐term strategy.

An overall episode for combined payment and quality assessment would require quite detailed measures. For example, in breast cancer, one would need tumor stage and its aggressiveness, as well as patient preference‐sensitive indicators such as fear of recurrence that may lead to a request for contralateral prophylactic mastectomy (CPM). CPM may not increase life expectancy, but it may impact the patient's perceived quality of life. A provider payment strategy should be neutral with respect to *patient* preferences; the clinicians helping a woman decide about CPM should not be compensated more (or less) based on her choice. The counselor might be paid on an hourly basis, with separate episode‐based payments for the chemotherapy and surgical/reconstruction teams.

- Research is needed regarding the practical issues in applying such an approach. Are claims data “good enough” for developing episodes, or is EHR data necessary?
- Is currently available episode software sufficiently well‐developed and transparent to be acceptable to providers and payers?
- Can one define episodes in a meaningful way for patients with multiple chronic conditions and, if not, what proportion of care would be omitted from an episode‐based model?
- What is the appropriate balance between fine distinctions among types of episodes (essentially risk adjustment) and the potential for gaming?
- From a payer perspective, are episodes better or worse than FFS for avoiding fraud and abuse, or for encouraging high quality?^7^

## Transitioning from the Current Set of Incentives

Economists and policy makers have long been dissatisfied with FFS. Although physicians are increasingly entering into employment‐based relationships, it is rare for their compensation to be based simply on time. No single compensation model, however, may be optimal for all situations. A mix of approaches applied selectively to different patient conditions or types of physicians, however, may be most likely to enhance value. It is far easier to demonstrate that a new payment model intended for just a subset of conditions/providers can benefit the physicians to whom it would apply while simultaneously increasing value. Willingness to accept an alternative requires overcoming physician uncertainty about how well the new approach will actually work and the expected transition costs associated with any change.

- There are many issues key to designing a mixed payment strategy about which we know relatively little. For example, which specific modes of payment, for example, per minute, per service, or per episode, would be most appropriate for specific types of clinical care or patient problems?
- Analyses should focus not on the amount paid, but on aligning the unit of payment with the choices to be made by the decision maker. Some argue that surgeons are overcompensated and that FFS leads them to be overly enthusiastic about the benefits of surgery. If, however, a patient's decision to have a procedure were informed by someone paid by the hour, would a FFS payment to the surgeon be problematic? Would an episode‐based payment be better?
- Such research should focus on the extent to which the physician being paid has discretion in determining the number of “units of work done” and clinical decision making responsibility for other costs incurred. Both the anesthesiologist and radiologist sell access to their skills. The anesthesiologist has little control over the time spent for a given procedure, and the radiologist has little control over the number of images ordered. This suggests their current per minute and per image payment models, respectively, may be reasonable.
- Would the answers to these questions be different for private payers who are not able to set prices the way Medicare can?

A minimal intervention approach to changing physicians' incentives might begin with relatively minor modifications to FFS payment to mitigate some of its most serious problems. For example, asking PCPs to accept salary or the risk associated with episode‐based payment may be difficult, especially in the context of changing compensation within a medical group. If evaluation and management (E&M) visits had a time‐based component, would this reduce physicians' disincentive to spend time to help their patients make better informed decisions?

- Research is needed on whether partially basing E&M visit payments on *time spent* with patients, rather than requiring documentation supporting the “complexity” of the visit would yield better value. Such research should explore various ways to modify the E&M codes, for example, giving credit for longer scheduled visits or allowing an “upcode” for an unplanned extension of a visit.
- In a similar vein, how should payment be offered for previsit electronic or telephone consultations that may substitute for face‐to‐face visits?
- Such research should consider not just the appropriate dollar amounts (not too little nor too much) but also the feasibility of monitoring such charges to minimize the potential for fraud and abuse.

Economists usually assume that “producers” operate to minimize their production costs. Physicians, however, rarely know how the resources used for their patients compare with those for patients seen by other physicians. Without the political and technical problems of putting physicians at financial risk with episode‐based payment, one could simply provide data to physicians on the costs incurred by their patients in specific episodes of care relative to those of other physicians. Berenson and Rice (2015) offer some provocative ideas that do not rely on specific financial incentives while Roland and Dudley (2015) offer suggestions on research regarding reputational reporting.

- Research is needed on whether feedback of information on relative cost patterns, without additional financial incentives, could alter physician behavior.
- How effective are other “behavioral nudges” in altering ordering patterns, for example, making resource conservative care the default option in EHR prompts?
- Are such approaches more effective if accompanied by agreed‐upon quality metrics to offset concerns about “skimping”?
- Does it matter if the feedback is given “privately” (physicians being labeled by code) versus “department publicly” (names known to all within the department) versus open to the public at large?
- Is there evidence that removing some of the current strong financial incentives while providing more information and encouraging professionalism will increase value?

Various aspects of medical practice may affect the adoption or impact of alternative payment models, for example, malpractice, board certification, scope of practice rules, maintenance of certification, adoption of electronic health records, and organizational context and supports. Physicians often claim they order tests that may not be truly necessary because a “missed finding” may increase their malpractice risk. Behavioral economics suggests people respond much more to a small probability of a large loss than to its actuarially equivalent gain. Thus, unless the economic rewards to alter one's behavior are very substantial, payment incentives perceived to increase the physician's malpractice risk may be resisted.

- Research is needed on whether physician ordering of tests for which they do not receive direct compensation is lower in states with lower rates of malpractice claims.
- Do state differences in scope of practice laws or ownership opportunities explain variations in the use of certain services?
- What strategies have proven effective in overcoming practice inertia?

## Conclusion

There is both substantial need and many opportunities to improve our understanding of how to construct and implement better incentives for value in care delivery. The issues are complex and the data, methods, and measures needed to answer these questions are evolving, as is the delivery system. Simple dichotomies such as FFS versus salary are helpful only for beginning the discussion. Physicians practice in many different settings, the types of patients seen vary (by clinical circumstance, preferences, and financial incentives), and the quality and completeness of the data vary, so it is difficult to infer either what incentive‐related factors drive the behaviors observed today or how any changes might alter those behaviors for better or worse. Given the charge to suggest ideas to inform policy within 5 years and reasonable budgets, these research suggestions focus on using selected situations in which it is easier to identify the unique contribution of an incentive on behavior.

No single payment approach is likely to be optimal for all types of care, patients, or settings. There may be specific roles for modified FFS, salary, and episode‐based payment models. Such hybrid approaches have important implications for the data systems and metrics required to support their implementation. The challenges, moreover, differ for changes at the national and organizational levels. National policy change should be value‐enhancing from a societal level and is typically constrained to be budget neutral in terms of federal dollars. The challenge is to design alternatives to the current system that meet those criteria, can survive Washington politics, and if voluntary, are sufficiently attractive that enough physicians and organizations choose to make the change. People and organizations tend to resist change, even when it is clearly advantageous, and the resistance is greater when the prospective advantages have yet to be proven in “situations like their own.”

At the organizational level, the challenges for suggesting alternatives are even more complex. Many HMOs and independent medical groups have been successful using various internal compensation strategies, but little is known outside those entities about the details of those arrangements and how they interdigitate with their internal countervailing performance metrics. Whatever is currently in place in each entity has a specific history—would those organizations rebuild their internal structures exactly as they are, or are they indeed currently seeking to make changes to better adapt to changing external environments? What are the internal challenges faced by organizations in making such changes? Even if the organization is more likely to succeed following a new strategy, there are certainly internal winners and losers, just as there will be at the national level. We need information not just on the best “end state,” but also on how to manage the transitions. As new organizations are developing in response to national policy changes, they need whatever lessons can be drawn from existing systems to be examined carefully to inform the design of better incentives to improve value. Well‐targeted research can help provide the evidence to move forward.

## Notes

1 In this paper, the term “physician” indicates an individual with decision‐making power in the care of patients. Most of the examples relate to physicians, but as used here, the term encompasses psychologists, advanced practice nurses, and the similar health professionals who can decide what they do and what tests, etc. they can order for patients. “Provider” is used to include entities such as hospitals that may be paid for certain medical care services. Such entities may employ clinicians or may merely serve as the site in which clinicians practice.

2 The usual episode grouper software uses claims as the data source, but pseudoclaims can be generated from an EHR, for example, an unbilled consultation by phone or e‐mail with an approximated relative value unit (RVU) attached. Assuming the grouper works well across the providers and patients in the study dataset, one can attach multipliers to each service (including the pseudoclaims) and get relative resources used in the episode. Although not valid across delivery systems, this may be good enough for internal comparisons *within* a research study.

3 The standard FFS payment model is based on relative value units (RVUs) multiplied by some dollar amount. RVUs, in turn, have several components. The physician work effort, or wRVU component, is supposed to reflect the physician work effort required to deliver the service. Additional components of the total RVU (tRVU) reflect malpractice liability costs and practice expense. In addition, there may be facility fees associated with certain services. There are typically extra charges for chemotherapy agents and their administration. Not all that revenue is necessarily passed on to the ordering oncologist, thus providing an opportunity to examine these incentive issues.

4 Ownership of lab facilities by primary care physicians (PCPs) may create similar incentives for overtesting, but the risk adjustment needed to account for PCP case mix can be quite complex.

5 We focus here on things that nearly always go together, not the services that may be possibly provided during the longer episode of care. See the discussion below.

6 Mangione‐Smith et al. (1999) examined this last question and found little support for this claim, but Linder et al. (2014) found that inappropriate antibiotic prescribing is more common toward the end a physician's shift.

7 See, for example, Geruso and Layton 2015, *NBER Working Paper*, “Upcoding: Evidence from Medicare on Squishy Risk Adjustment.”
